# Estimation of Total Hemoglobin (SpHb) from Facial Videos Using 3D Convolutional Neural Network-Based Regression

**DOI:** 10.3390/bios15080485

**Published:** 2025-07-25

**Authors:** Ufuk Bal, Faruk Enes Oguz, Kubilay Muhammed Sunnetci, Ahmet Alkan, Alkan Bal, Ebubekir Akkuş, Halil Erol, Ahmet Çağdaş Seçkin

**Affiliations:** 1 Department of Electrical and Electronics Engineering, Osmaniye Korkut Ata University, 80000 Osmaniye, Türkiye; ufukbal@osmaniye.edu.tr (U.B.); kubilaysunnetci@osmaniye.edu.tr (K.M.S.); halilerol@osmaniye.edu.tr (H.E.); 2 Hassa Vocational School, Hatay Mustafa Kemal University, 31060 Hatay, Türkiye; farukenes.oguz@mku.edu.tr; 3 Department of Electrical and Electronics Engineering, Kahramanmaraş Sütçü İmam University, 46050 Kahramanmaraş, Türkiye; 4 Department of Pediatrics, Manisa Celal Bayar University, 45140 Manisa, Türkiye; alkan.bal@cbu.edu.tr; 5 Germencik Yamantürk Vocational School, Aydın Adnan Menderes University, 09010 Aydın, Türkiye; ebubekir.akkus@adu.edu.tr; 6 Department of Computer Engineering, Aydın Adnan Menderes University, 09010 Aydın, Türkiye

**Keywords:** 3D residual CNN, deep regression, non-contact SpHb, non-invasive monitoring, total hemoglobin, facial video analysis, medical imaging AI

## Abstract

Hemoglobin plays a critical role in diagnosing various medical conditions, including infections, trauma, hemolytic disorders, and Mediterranean anemia, which is particularly prevalent in Mediterranean populations. Conventional measurement methods require blood sampling and laboratory analysis, which are often time-consuming and impractical during emergency situations with limited medical infrastructure. Although portable oximeters enable non-invasive hemoglobin estimation, they still require physical contact, posing limitations for individuals with circulatory or dermatological conditions. Additionally, reliance on disposable probes increases operational costs. This study presents a non-contact and automated approach for estimating total hemoglobin levels from facial video data using three-dimensional regression models. A dataset was compiled from 279 volunteers, with synchronized acquisition of facial video and hemoglobin values using a commercial pulse oximeter. After preprocessing, the dataset was divided into training, validation, and test subsets. Three 3D convolutional regression models, including 3D CNN, channel attention-enhanced 3D CNN, and residual 3D CNN, were trained, and the most successful model was implemented in a graphical interface. Among these, the residual model achieved the most favorable performance on the test set, yielding an RMSE of 1.06, an MAE of 0.85, and a Pearson correlation coefficient of 0.73. This study offers a novel contribution by enabling contactless hemoglobin estimation from facial video using 3D CNN-based regression techniques.

## 1. Introduction

The hemoglobin (Hb) disorders constitute a serious health problem in 71% of 229 countries worldwide, accounting for 89% of global births. More than 330,000 infants are affected annually, including 83% with sickle cell disorders and 17% with thalassemia. Hb disorders account for approximately 3.4% of deaths in children under 5 years of age [1]. Globally, it is estimated that 40% of all children aged 6–59 months, 37% of pregnant women, and 30% of women 15–49 years of age are affected by anemia. Anemia caused 50 million years of healthy life loss due to disability in 2019. The largest causes were dietary iron deficiency, thalassemia and sickle cell trait, and malaria [2]. Hb is vital in the diagnosis of diseases such as severe infection, trauma, hemolytic diseases, and favism. Current Hb measurements require invasive blood sampling and blood analysis, which is a time-consuming method [3].

Hb, a protein in red blood cells, plays a crucial role in transporting and distributing oxygen throughout the body, especially in conditions involving hemolysis and trauma. Rapid assessment of Hb levels is essential to enhance treatment efficacy and reduce response time in serious Hb-related conditions [4]. Diseases such as anemia and thalassemia require frequent monitoring of blood Hb levels for effective diagnosis and treatment. Patients typically undergo blood tests every 3–4 weeks, with transfusion decisions made as needed. This process is not only time-consuming but also involves uncomfortable, often painful procedures for patients [5].

Oximeters are non-invasive devices that measure and display parameters like oxygen saturation and pulse. In addition to enhancing oxygen saturation measurement, they also detect different Hb types. However, current oximeters require skin contact, which limits their use in cases of open wounds, cold extremities, certain medical conditions, or compromised tissue integrity, where probe-based measurements may not be feasible and can lead to inaccurate results [6,7]. Due to these limitations, there has been a recent increase in research on non-invasive, non-contact methods for detecting total hemoglobin (SpHb). The objective of this study is to develop a non-contact method for detecting SpHb using three-dimensional regression models applied to facial videos collected from volunteers. Facial videos include not only spatial details like skin color and shape but also temporal changes over time. These small changes, such as blood flow under the skin, happen in a few seconds and cannot be seen clearly in a single image. By using video, it becomes possible to track these changes frame by frame. This gives more useful information to the model. For this reason, videos help the model estimate SpHb values more accurately than single RGB images. Hb levels were measured using a Masimo device, resulting in a labeled dataset employed for the development and comparison of three distinct types of three-dimensional regression models: three-dimensional CNN (3D CNN) (baseline), channel attention-enhanced 3D CNN, and residual 3D CNN. Furthermore, all models were integrated into a user-friendly graphical user interface (GUI) application, enhancing accessibility and usability.

The following sections of the paper are organized as follows: Section 2 presents a review of the existing literature on the topic and summarizes the contributions of this study. Section 3 provides comprehensive information about the dataset used in the study, the proposed models, the performance metrics used, and the GUI developed. Section 4 presents the findings from the experimental results and discussions, while Section 5 provides conclusions, concluding remarks, and future research.

## 2. Related Works

In this section, existing studies in the literature for non-contact SpHb as well as anemia detection and estimation using machine learning (ML) and deep learning (DL) techniques are reviewed.

Mobile technology has high potential usage because it provides a quick, accessible, and non-invasive method for anemia detection, particularly in resource-limited settings. For rapid anemia prediction, mobile application-based studies come to the fore.

Suner et al. [8] developed a smartphone-based method to estimate Hb concentration and screen for anemia using conjunctiva images from 344 patients. The study achieved accuracy rates of 82.9% for Phase 1 and 72.6% for Phase 2. While the researchers noted that image quality improvements could enhance hemoglobin prediction, the accuracy for anemia detection was limited at 73%, which may impact the method’s clinical applicability. The study also faced challenges due to a small dataset of 142 samples, lack of standardized image acquisition procedures, and the requirement for specific patient positioning, which could limit real-world usability. Additionally, varying lighting conditions were not considered, further constraining the method’s generalizability.

Mannino et al. [9] developed a smartphone application to detect anemia by analyzing images of the nail bed captured by a smartphone. The app estimates Hb levels by evaluating the color and metadata of these images, offering a non-invasive method for anemia detection. However, the study’s dataset is limited to 50 and 100 test samples, which may restrict the generalizability of the findings across diverse populations and varying lighting conditions. Additionally, the app faces challenges in accurately measuring Hb levels when nail abnormalities, such as leukonychia, nail injuries, or discoloration from nail polish and medication, are present. It also requires manual filtering of unclear or faded nail bed images, which can be time-consuming and subject to human error.

Appiahene et al. [10] conducted a study to detect anemia using palm images, comparing various ML models, including convolutional neural network (CNN), k-nearest neighbor, decision tree, naïve Bayes, and support vector machine. The initial dataset comprised 527 images, which was subsequently expanded to 2635 images using data augmentation techniques. The naïve Bayes model demonstrated the highest accuracy, achieving a score of 99.96%. While the study effectively utilized data augmentation to enhance the dataset, applying it to all images, including those in the training, validation, and test sets, may have influenced the reported results by introducing a risk of overfitting. The test set evaluates a model’s performance on unseen, real-world data, so it should remain untouched to maintain its representativeness. In addition, data augmentation should only be applied to the training set to improve the robustness of the model without introducing bias. Additionally, the study focused solely on anemia classification without performing a SpHb estimation.

Moral et al. [7] explored the potential of DL techniques for estimating SpHb concentration. The study employed transfer learning with AlexNet, a pre-trained CNN, to analyze data collected using a custom-designed frequency-domain multi-distance (FDMD) non-contact oximeter. While the model demonstrated promising test accuracies of up to 87.50% for both 20 and 50 MHz frequencies, the sophisticated and costly nature of the data acquisition setup limits its practicality in real-world clinical settings. Furthermore, although the reported accuracy rates are encouraging, they may not meet the stringent standards required for clinical diagnosis and treatment.

Zhang et al. [11] conducted a study utilizing a dataset of video recordings from 316 patients. The study aimed to develop a ML system supported by video extraction and face recognition methods to detect anemia status in males and females, as well as to identify mild and severe anemia cases. The study reported accuracy rates of 84.02%, 70.58%, and 68.42% for these tasks, respectively. However, the study only classified anemia and did not perform SpHb estimation. The dataset excluded white and black skin tones, limiting the model’s generalizability to individuals with diverse skin tones. Additionally, the model struggled to accurately detect mild and severe anemia cases. The study did not account for variations in lighting conditions and relied on a high-resolution camera with time-intensive measurements, such as 45-degree rotations, making the approach impractical for routine clinical use. Furthermore, the model’s low specificity (around 67%) indicated a tendency to misclassify non-anemic individuals as anemic.

Zhao et al. [12] developed a DL model that leverages ultra-widefield (UWF) fundus images to predict Hb concentrations and screen for anemia. The model achieved a MAE of 0.83 g/dL for Hgb prediction and an area under the receiver operating characteristic curve (AUC) of 0.93 for anemia screening. The study emphasized the role of retinal features, such as the optic disc and retinal vasculature, in anemia prediction. Despite its promising outcomes, the model faced challenges, including the high cost of acquiring UWF images, which hinder its real-time detection and broad clinical application. Additionally, the model’s accuracy was influenced by various disease conditions. Future research is needed to address these limitations and investigate the model’s potential for screening other health conditions using a single UWF image.

Yılmaz et al. [13] proposed a deep learning approach that integrates numerical data (e.g., age and body mass index) with nail bed images to non-invasively estimate Hb levels. They employed a dual-branch architecture, consisting of a CNN to extract features from 128 × 128 nail bed images and a multilayer perceptron (MLP) to process the numerical data. The outputs of these two branches are merged and fed into a final regression layer to predict Hb levels. The model achieved a mean absolute percentage error (MAPE) of 2.09% and a root mean square error (RMSE) of 0.56 g/dL. While these results are promising, several practical and methodological limitations must be addressed before clinical deployment. Training the model for 1000 epochs on a small dataset of just 247 subjects poses a risk of overfitting, potentially hindering its ability to generalize to unseen data. One limitation stems from the controlled lighting conditions used during data collection: all images were captured inside a closed box covered with black felt using a fixed flash. This makes the model highly sensitive to deviations from those conditions, potentially reducing its performance in real-world environments. Another important constraint is the strict requirement for clean, natural nails; the presence of cosmetic products such as nail polish or artificial nails, as well as color changes caused by trauma or disease, can adversely affect prediction accuracy and limit usability in daily life.

Appiahene et al. [14] proposed a ML-based model for pallor analysis and anemia detection using 710 conjunctival eye images. The model, which integrated CNN, logistic regression, and Gaussian fuzziness algorithms, utilized YOLOv5 for automatic detection and extraction of the conjunctiva, which was then used to create a region of interest (ROI). The resulting model achieved impressive performance metrics, including 90% sensitivity, 95% specificity, and 92.5% accuracy, with results obtained within approximately 50 s. This model was integrated into a smartphone application for practical use in anemia detection. Despite the promising results, the study highlighted several limitations. The use of smartphones with low-quality cameras (below 5 MB) could affect the image quality, ultimately impacting the accuracy of the model. Furthermore, weak internet connection influenced the application’s testing duration, limiting its reliability under certain conditions. The study was also constrained by its focus on children aged 6 to 59 months, making the findings less applicable to other age groups or broader populations. Another challenge noted was the need for manual intervention, such as pulling the lower eyelid to capture conjunctiva images, which may affect the method’s practical usability. Additionally, the study did not specify a gold standard for validation or provide detailed information on the validation process. Finally, the authors emphasized the importance of applying data augmentation only during training, as doing so during testing could undermine the model’s generalizability to real-world data.

Hu et al. [15] introduced a promising non-invasive method for Hb monitoring using smartphone eye images. This approach integrated a mask region-based convolutional neural network (R-CNN) for segmentation and MobileNetV3 for regression, yielding a coefficient of determination value of 0.503 and a MAE of 1.6 g/dL, suggesting reasonable accuracy in estimating Hb levels. Despite the promising results, the study had notable limitations. The dataset was relatively small and imbalanced, with more images from non-anemic patients, which could have influenced the model’s performance. Additionally, the research was conducted in a single center, limiting the generalizability of the findings. To improve model robustness, the authors recommended enlarging the dataset, conducting external validation across multiple centers, and addressing potential biases by incorporating data from standard venous analyzers

Chen et al. [16] developed a smartphone-based system that employs DL algorithms to predict non-invasive Hb levels by analyzing images of the eyelids. The system incorporates several models, including efficient group enhanced UNet (EGE-UNet) for the segmentation of eyelids and delta Hb adaIN (DHA (C3AE)) for the prediction of Hb levels. The system demonstrates considerable promise, achieving performance metrics including a mean absolute error of 1.34, mean squared error of 2.85, root mean square error of 1.69, and coefficient of determination of 0.34. Nevertheless, despite these encouraging outcomes, the study underscores the necessity for further optimization to attain the requisite level of accuracy for clinical applications. Additionally, the authors identify several limitations, including a relatively modest sample size, inconsistencies in smartphone camera quality, and environmental factors that could potentially influence the accuracy of the system. These challenges may restrict the applicability of the findings and addressing them is crucial for ensuring the system’s robustness and reliability in diverse real-world settings.

Recent advancements in non-invasive Hb estimation and anemia detection have employed various innovative approaches. Chen et al. [16] developed a smartphone-based system that captures eyelid images to predict Hb levels using DL algorithms, offering a portable solution for real-time assessments. Lin et al. [17] introduced the Body-Part-Anemia Network (BPANet), which utilizes images from the conjunctiva, palm, and fingernail to estimate Hb concentration, demonstrating the effectiveness of multi-site imaging. Mitani et al. [18] focused on retinal fundus images, employing DL to detect anemia and quantify Hb levels, highlighting the potential of ocular imaging in hematological assessments. Khan et al. [19] also leveraged retinal images, developing a scalable DL model suitable for resource-limited settings to detect anemia and estimate Hb levels. Chen et al. [20] proposed a non-invasive measurement system based on four-wavelength photoplethysmography (PPG), combining optical sensors with ML to accurately predict Hb concentration.

In our proposed method, except for the methodological differences mentioned above, the exact Hb estimation is made only from the face area. Additionally, several prior studies are limited by small dataset sizes, with sample counts ranging from 31 to 104 individuals [21,22,23]. In addition, in some studies, the test dataset was also subjected to data augmentation [10,14]; therefore, biased high accuracy rates were obtained. Moreover, unlike the method we proposed, the estimation process was performed with the help of a single image instead of a video recording [8,9,10]. Some studies have focused solely on categorical classification of anemia using predefined Hb thresholds, without attempting to estimate continuous Hb concentration values [24]. Non-standard (requiring intervention) data collection methods were applied in some studies, and this may limit the practical applicability of the method and make it difficult to use in real application conditions [8,9]. In addition to this, there are deficiencies in studies that combine both spatial and temporal information and consider long-range spatiotemporal relationships. In this study, SpHb is estimated from raw facial videos with different lighting conditions using 3D CNN-based regression models. Herein, no standardization method is needed for emergencies. Additionally, this study does not utilize special preprocessing techniques such as denoising, contrast enhancement, and segmentation. Based on all this information, the proposed method provides innovative and competitive advantages compared to literature.

As can be seen in the literature review, researchers have addressed the issues of Hb level detection/estimation and have used various DL methods in different studies in this direction. Unlike the studies in the literature, SpHb detection was performed in this study using three different 3D CNN-based regression models. In addition, to train these models and measure their performance, facial video recordings of 279 different volunteers were collected, and an original dataset was obtained. The models were trained using the dataset obtained within the scope of the study, and the performances of the models were tested using RMSE, MAE, MSE and Bland–Altman Plot metrics. In addition, a user-friendly GUI was developed for these models. To the best of the authors’ knowledge, this study is the first to directly estimate SpHb from facial videos using a 3D CNN based video regression model.

The contributions of this study can be highlighted as follows:

- Baseline, channel attention-enhanced, and residual 3D CNN based regression models were developed for SpHb detection.
- A unique dataset that can be used for SpHb detection was obtained by collecting 30-s facial video images from 279 volunteers.
- The three different models developed were applied on the test data and the comparative results of the models were presented.
- A user-friendly GUI was developed for the use of trained models.

## 3. Materials and Methods

### 3.1. Dataset

The experiments utilized the integrated webcam of a Lenovo LOQ 15IRH8 laptop (Lenovo, Beijing, China). All videos were recorded in color (24-bit RGB with three channels × 8 bits/channel) at 30 frames per second (fps) with a pixel resolution of 1280 × 720 and saved in MP4 format. The illumination source was either indirect sunlight or fluorescent lighting. Each session lasted 60 s, during which healthy volunteers were positioned approximately 50 cm from the webcam. Videos were chosen instead of single images for three reasons. First, facial videos include small color changes over time due to blood flow, which can be used as a signal similar to rPPG in medical devices. Second, by analyzing many frames, random noise such as lighting changes or small movements can be reduced. Third, videos allow continuous tracking of hemoglobin trends, just like Masimo’s SpHb monitors. With our 3D CNN models, these spatial and temporal features help improve accuracy in a contactless and practical way. A total of 279 experiments were conducted indoors to evaluate the SpHb assessment method. To validate the results, SpHb values obtained with a Masimo oximeter were used as a reference.

**Figure 1:**
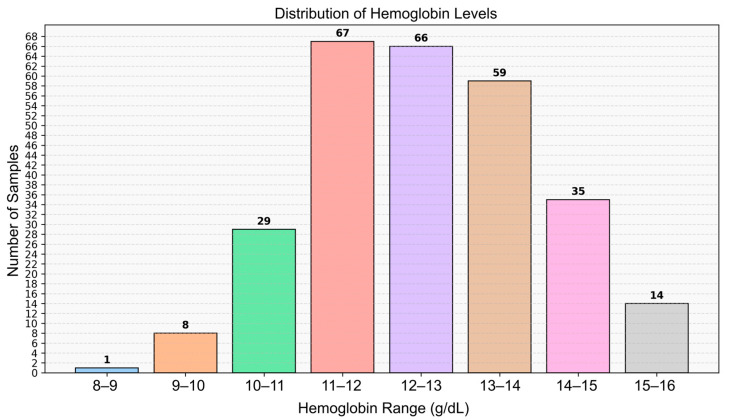
Histogram showing the distribution of Hb levels among participants.

**Figure 2:**
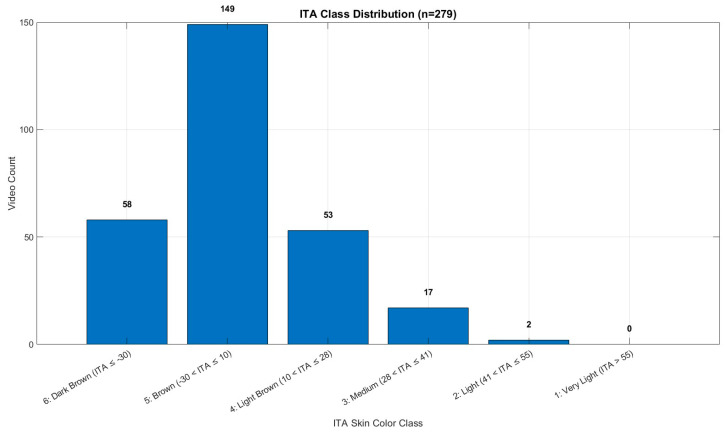
ITA class distribution.

**Figure 3:**
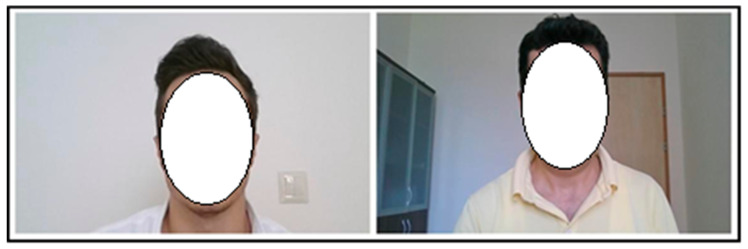
Sample frames from the dataset used in the study.

The study population consisted of 279 healthy volunteers, including 126 males and 153 females, from whom video data were collected. The mean Hb concentration was 12.48 g/dL, with a range of 8.90 to 15.80 g/dL. All recordings were made indoors during daytime hours using only ambient natural light from windows (indirect sunlight), without any artificial illumination. This approach was intended to ensure a consistent and realistic lighting environment. The dataset comprises healthy young adult volunteers from Türkiye; therefore, skin tone diversity may be limited due to the regional population characteristics. Ethical approval was obtained prior to the commencement of the study (Manisa Celal Bayar University, Faculty of Medicine, Health Sciences Ethics Committee, Date: 10 July 2024 Decision No: 20.478.486/2515). The details of the dataset used in the study are shown in Figure 1. The number of individuals below and above the anemia threshold (12 g/dL) is provided in Table 1. The distribution of skin tone categories based on ITA classes is shown in Figure 2, and representative sample frames from the dataset are illustrated in Figure 3.

### 3.2. Regression with 3D CNN Methods

Although CNNs are generally used for classification tasks, they can also be used for regression tasks. The process of converting a classification network to a regression network consists of four stages: replacing the last full connection layer, removing the softmax layer, updating the loss function, and direct regression estimations [25,26,27]. In addition, 3D CNNs can analyze both spatial and temporal features of a video at the same time. Because of this, CNNs will be used for video classification and regression tasks [28].

This study employs three 3D CNN architectures to perform regression on facial video inputs and directly estimate a continuous Hb level. Unlike conventional 2D CNNs, 3D CNNs jointly capture spatial and temporal features from video data, making them well-suited to detect subtle skin tone and blood flow changes correlated with Hb levels [24].

To convert each network from classification to regression, the final softmax layer is replaced with a single-unit regression output.

- Baseline 3D CNN Model: A compact architecture serving as a performance baseline.
- Channel Attention-Enhanced 3D CNN Model: A deeper network augmented with channel attention modules, which emphasize Hb-related visual cues, such as subtle skin-tone and blood-volume changes, while suppressing noise.
- Residual 3D CNN Model: A ResNet-inspired 3D CNN that uses residual connections to facilitate training of a deeper network by mitigating vanishing gradient issues.

**Figure 4:**
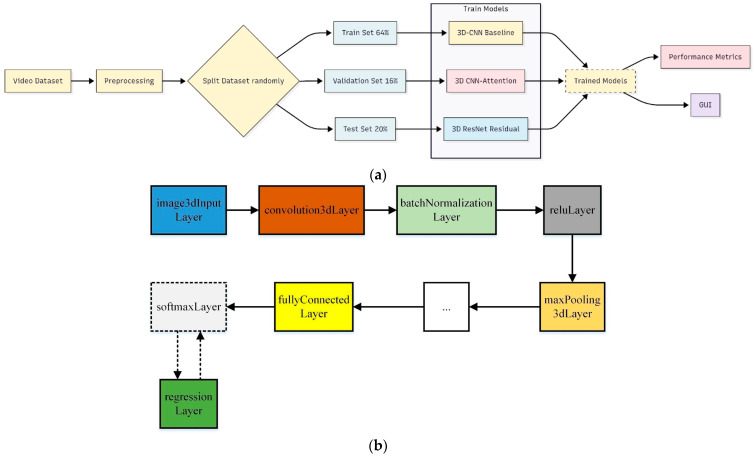
Block diagrams of the methods proposed in the study. (**a**) Training of developed models. (**b**) Converting classification network into regression network.

Before training, each original recording (30 s^–1^ min at 30 fps) was sampled to a fixed length of 224 frames, and every frame was resized to 224 × 224 pixels to ensure uniform input dimensions. The full dataset of 279 videos was first split 80:20 into training and testing sets, and the resulting 80% training portion was then split 80:20 again into training and validation, yielding 64% training, 16% validation, and 20% test sets. All three models were trained on the training set, tuned on the validation set, and evaluated on the held-out test set. The top-performing model was subsequently integrated into a user-friendly GUI. A block diagram of this workflow is shown in Figure 4.

#### 3.2.1. 3D CNN (Baseline) Regression Model

**Figure 5:**
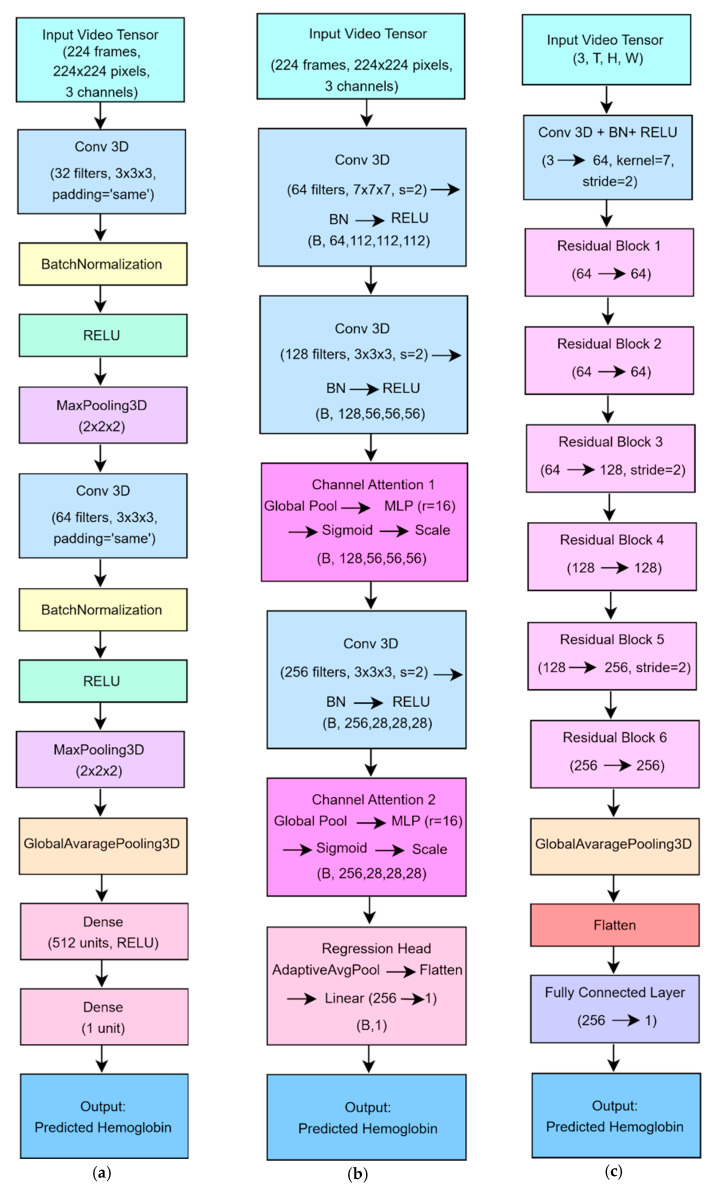
Architecture of DL models (**a**) The baseline 3D CNN model for Hb estimation. (**b**) The channel attention-enhanced 3D CNN model. (**c**) The residual 3D CNN model.

A simple 3D CNN which is shown in Figure 5 a served as a baseline to regress Hb concentration from facial video sequences [29]. The network ingested fixed-length clips of 224 RGB frames at 224 × 224 pixels. Its architecture comprised two convolutional blocks: each block began with a 3 × 3 × 3 Conv3D layer (padding = “same”), including 32 filters in the first block and 64 in the second, followed by batch normalization, ReLU activation, and 2 × 2 × 2 MaxPooling3D to halve the spatial and temporal dimensions. A GlobalAveragePooling3D layer then collapsed the resulting spatiotemporal feature maps into a single 1D vector. This vector was passed through a fully connected layer of 512 units with ReLU activation and finally through a single-unit output layer to produce the Hb estimate.

The model was trained to minimize mean squared error using the Adam optimizer (learning rate = 1 × 10^−3^, batch size = 4) for up to 100 epochs on an NVIDIA RTX-6000 GPU. This compact architecture offered low computational overhead and rapid convergence, making it an effective baseline for comparison against the deeper residual model.

#### 3.2.2. Channel Attention-Enhanced 3D CNN Model

CNN architectures typically have an increasing number of channels, and these channels can be thought of as different perspectives in feature extraction. Channel attention mechanisms are used to detect relationships between channels and allow the model to prioritize the most relevant channels [29,30]. The model architecture is shown in Figure 5 b. This model extends the baseline architecture by incorporating channel attention modules to enhance the network’s ability to focus on Hb-relevant features during regression. The model leverages 3D convolutions to jointly extract spatial and temporal information from facial video sequences. These features include subtle skin tone variations, structural cues, and dynamic changes across consecutive frames. To further improve performance, channel attention blocks are introduced to adaptively reweight feature channels, allowing the model to emphasize informative signals, such as those associated with remote photoplethysmography (rPPG), while suppressing irrelevant or noisy inputs. This attention mechanism enhances the model’s sensitivity to minor blood volume fluctuations, which are indicative of Hb concentration. As a result, the model achieves improved prediction accuracy and interpretability, particularly under real-world conditions involving motion or varying illumination. Additionally, its structure supports real-time operation, making it suitable for practical, non-contact Hb monitoring.

#### 3.2.3. Residual Regression Model

A model for predicting Hb concentration from facial video clips was developed using a 3D ResNet-based convolutional network [29,31]. The architecture is shown in Figure 5 c. The network accepts a fixed-length input of 224 RGB frames at 224 × 224 pixels. Its architecture begins with an initial feature-extraction block composed of a 7 × 7 × 7 convolution (stride 2, padding 3), followed by batch normalization and ReLU activation to capture low-level spatiotemporal features while halving each spatial dimension. The core of the model comprises six residual blocks, each built around two 3 × 3 × 3 convolutions (with Batch Normalization and ReLU) plus a shortcut connection that either performs identity mapping or projects dimensions via a 1 × 1 × 1 convolution. The first two blocks maintain 64 feature channels. The third and fourth blocks expand to 128 channels, with the third block also down sampling via stride 2. The fifth and sixth blocks expand to 256 channels, with the fifth block likewise performing down sampling. This residual structure mitigates vanishing gradients and accelerates learning in deep layers. After the last residual block, an adaptive global average pooling layer reduces each 3D feature map to a single scalar, thereby decreasing parameter count and reducing overfitting risk. A final fully connected layer then projects the resulting 256-dimensional vector to a single continuous output, yielding the Hb estimate. The model was trained using mean squared error loss and the Adam optimizer (learning rate = 1 × 10^−3^, batch size = 4) for up to 100 epochs on an NVIDIA RTX-6000 GPU.

### 3.3. Performance Metrics

This section describes the performance metrics used in the study. These metrics are root mean squared error (RMSE), mean absolute error (MAE), coefficient of determination (R^2^), Pearson correlation coefficient (PCC), and mean squared error (MSE). It is useful to analyze these metrics to understand whether the proposed models can predict successfully. Based on this information, descriptions of RMSE, MAE, MSE, MAPE, R^2^, PCC, and MSE metrics as well as Bland–Altman Plot are given in the following paragraphs.

This metric, called RMSE or root mean squared deviation, is equal to the square root of the mean of the squares of all errors and expresses the standard deviation of those errors. The formula of the RMSE metric used in the study can be given as follows [32]:

(1) $$\sqrt{\frac{1}{k}\sum_{i=1}^{k}{\left(x_{i}-y_{i}\right)}^{2}}$$

MAE gives the average of the absolute difference between the actual and predicted values, and a generic and bounded performance measure for the model is provided if outliers represent the corrupted part of the data. Herein, the formula for the MAE metric can be written as noted in the following equation [32,33]:

(2) $$\frac{1}{k}\sum_{i=1}^{k}\left|x_{i}-y_{i}\right|$$

MSE is a metric commonly used to find the squared difference between actual and predicted values, and this metric indicates how close the line of best fit is to the point set. The formula of this metric can be provided as noted in Equation (3) [32,34] where *k, x_i_*, and *y_i_* denote the number of predictions, actual values, and predicted values, respectively [32,33,34].

(3) $$\frac{1}{k}\sum_{i=1}^{k}{\left(x_{i}-y_{i}\right)}^{2}$$

MAPE is an error metric that measures how much a model’s predictions are wrong compared to the true values. It is widely used to evaluate the accuracy of predictions, especially in regression problems, and expresses the error rate as a percentage [35]. The equation of MAPE is presented in Equation (4) where k, $x_{i}$, $y_{i}$, and $\bar{y_{i}}$ denote the number of predictions, actual values, predicted values, and mean of the actual values, respectively.

(4) $$\frac{100\%}{n}\sum_{i=1}^{k}\left|\frac{x_{i}-y_{i}}{x_{i}}\right|$$

PCC or Pearson’s r is defined as a metric used in statistics to measure the strength of the relationship between two variables and their relationship to each other. In other words, the PCC calculates how much and in what direction one variable changes as the value of the other variable changes [36].

R^2^ can be defined as the proportion of the variance in the target variable that can be predicted by the characteristics (explanation rate). The closer this metric is to 1, the better the model will explain the variance in the target variable [33]. The equation of R^2^ is presented in Equation (5) where k, $x_{i}$, $y_{i}$, and $\bar{y_{i}}$ denote the number of predictions, actual value, predicted value, and mean of the actual values, respectively.

(5) $$1-\frac{\sum_{i=1}^{k}{\left(x_{i}-y_{i}\right)}^{2}}{\sum_{i=1}^{k}{\left(x_{i}-\bar{y_{i}}\right)}^{2}}$$

Bland–Altman plot (difference plot): A Bland–Altman (difference) plot is a method commonly used in analytical chemistry and biomedicine to evaluate the agreement between two tests. These plots are frequently employed to compare measurements from different instruments or techniques, allowing for the identification of potential systematic differences (e.g., constant bias) or outliers [37].

### 3.4. Graphical User Interface

**Figure 6:**
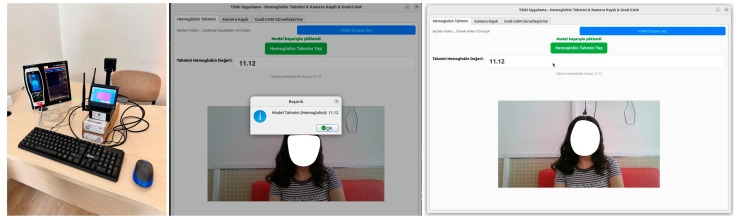
Screenshots of the GUI designed in the study.

A user-friendly GUI application developed to predict SpHb from facial videos is shown in Figure 6 and presented in this section [29]. This GUI application has three panels: Hb estimation, video recording, and Grad-CAM. Furthermore, an existing video file can be uploaded to the application with the help of a button. This application has a panel that allows video recording. For Grad-CAM visualization, users can easily switch between panels with a single click. Using the video upload button, the user can effortlessly upload the video to the user-friendly software. Thus, appropriate data input can be provided for the models trained in the study. On the other hand, the most successful model has been embedded in this user-friendly GUI application. Herein, users can use this most successful model effectively with the predict button. The SpHb value predicted by the most successful model developed in the study is presented to the user on the screen.

This user-friendly GUI application can be effortlessly closed with a button. Screenshots of the user-friendly software and video recording setup designed within the scope of the study are shown in Figure 6. It is necessary to note that all the models used in the study are tested with the same videos, and the video loaded to the GUI application in Figure 3 is also a test video. In this figure, the actual SpHb value predicted by the most successful model from the video used is equal to 11.12. With the predict button, users can obtain predicted values in approximately 2 s.

## 4. Results and Discussion

### 4.1. Experimental Results

**Figure 7:**
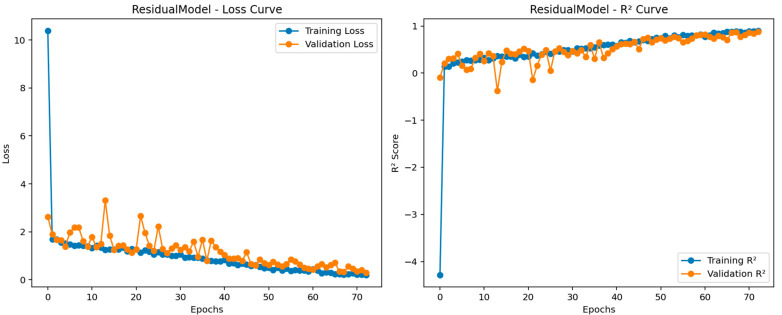
Training progress for the residual regression model.

In this section, the training parameters and Bland–Altman plots obtained from these models are presented for the proposed models. Additionally, training progress of the most successful model is also provided in this section. This section provides a better understanding of the results and discussion. For this purpose, the training parameters of the three video-based regression models used in this study are given in Table 2. Table 2 indicates the parameters chosen for the training of these models proposed in this study, and these parameters are the same for all three models. For three models, the optimizer is set to Adam. In addition, the epochs value determined for training the models is equal to 100. The batch size value used in the training of these models is 4. In light of this information, the training process of the most successful model developed is presented in Figure 7.

**Figure 8:**
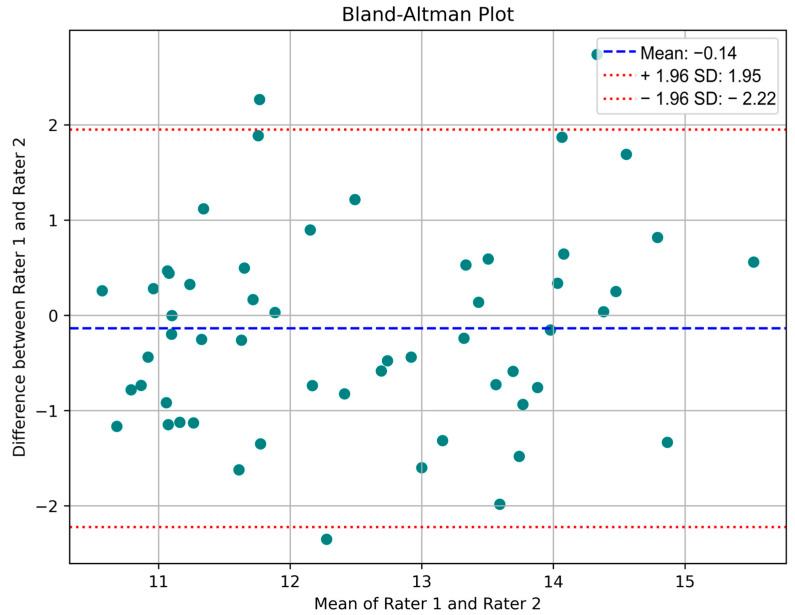
Residual regression model of Bland–Altman plot.

Figure 7 shows the training progress for the most successful model. Herein, the RMSE and loss values obtained per iteration for the most successful architecture developed can be seen im the figure. As mentioned before, since the total number of epochs is 50, and the total number of iterations per epoch is 6. The maximum iteration value is equal to 300. Given the frequency of validation for all models, the models are validated with a validation set at a specific iteration. The reason for changing the learning rate here is to ensure that the architectures are not subject to overfitting. The blue and orange points in this figure correspond to the training R2, training loss, and validation R2 and loss values, respectively. Moreover, the validation RMSE value obtained for the most successful regression model is equal to 1.2966. From this figure, it is seen that there is an absence of overfitting in the developed model. The Bland–Altman plots obtained from the test data of these trained models are presented in Figure 8. The Bland–Altman plot is a graphical tool used to compare the level of agreement between two different measurement methods. It plots the difference between the measurements against their mean, making it easy to identify systematic bias and random variation. Especially for the last model, we can conclude that the mean difference (bias) is close to zero, indicating no significant systematic bias, while most data points are within the limits of agreement, indicating acceptable prediction accuracy.

### 4.2. Discussion

This section presents evaluation metrics obtained from test set of the 3D CNN (baseline), channel attention enhanced 3D CNN, and residual 3D CNN regression models developed in this study. In addition, the controversial place of this study in the literature is also given in this section.

The evaluation metrics for the three models are summarized in Table 3, showing a clear, stepwise improvement in test set performance. The baseline 3D CNN yielded an RMSE of 1.7897, MAE of 1.4269, MSE of 3.2029, and PCC of 0.5744. Incorporating channel attention led to substantial gains. The channel attention 3D CNN achieved an RMSE of 1.1733, MAE of 0.9630, MSE of 1.3767, and PCC of 0.6563. The residual 3D CNN outperformed both, recording the lowest errors (RMSE = 1.0600, MAE = 0.8500, MSE = 1.1300) and the highest correlation (PCC = 0.7300), underscoring its superior accuracy and predictive strength for Hb estimation from facial videos. In the light of all this information, it is seen that the 3D-based regression methods developed in this study have competitive results according to the literature [8,9,10,11,12,13,14,15,16,17,18,19,20,21,22,23,24]. The fact that the training times of the 3D CNN-based regression models proposed in this study are reasonable enough allows them to be developed effectively. When the studies in literature are examined, it is generally possible to predict these values using images. In this study, the development of 3D CNN-based regression models using facial videos is one of the other contributions of the study. In addition, instead of using all video frames, the use of a specified size and number of frames provides an advantage in training and prediction time. The size and number of frames used in this study are equal to 224 and 30, respectively (224 × 224 × 3 × 30). In this study, the utilization of dropout layers at specified values prevents overfitting in the models developed for Hb prediction. Herein, the test performance metrics available for the models in the literature show this situation [8,9,10,11,12,13,14,15,16,17,18,19,20,21,22,23,24]. When the proposed method is analyzed, it is seen that the MAE value of 0.85 can be obtained in the residual model. When this value is compared with the results of the studies in Table 3, it is seen that the proposed method has successful performance metrics. In addition, a user-friendly GUI application containing the most successful model has been prepared in this study. With this GUI application, users can efficiently and effectively use the developed 3D CNN-based video regression model. This GUI application presents the predicted SpHb value to the users in a panel in about 2 s.

### 4.3. Comparative Analysis of Results and Methodologies

In recent years, several studies have explored non-invasive Hb estimation using DL techniques. For instance, Chen et al. developed a smartphone-based system that captures eye images and employs a deep neural network for Hb prediction, achieving a MAE of 1.34 g/dL [16]. Similarly, Lin et al. introduced a model utilizing images from the conjunctiva, palm, and fingernail, reporting an accuracy of 84.9% in anemia detection [17]. Mitani et al. focused on retinal fundus images, achieving an area under the receiver operating characteristic curve (AUC) of 0.88 for anemia detection [18]. In comparison, our study employs 3D CNNs to analyze facial videos, resulting in an MAE of 0.85 g/dL. These findings suggest that our approach offers comparable accuracy to existing methods while providing the added advantage of utilizing readily accessible facial videos, potentially enhancing user convenience and compliance. Recent studies presented in this section are compared in Table 4. It highlights the effectiveness of various non-invasive Hb estimation methods, and it can be said that there is a promising balance between accuracy and practicality that we achieved with the proposed method. As presented in Table 4, recent studies utilize a wide variety of input modalities and algorithmic models for non-invasive Hb estimation. The existing literature presents various approaches, such as [20], reporting lower MAE or higher classification accuracy. However, these methods often require dedicated PPG sensors or controlled imaging systems and were validated on relatively small cohorts (e.g., 58 subjects). Similarly, a notable study by [19] achieved a lower MAE of 0.58 g/dL compared to our 0.85 g/dL. Importantly, the data used by [19] also depended on specialized equipment, including the Carl Zeiss VISUCAMlite (Carl Zeiss, Jena, Germany) and Optos UWF devices OPTOS (Daytona; Optos PLC, Dunfermline, UK) for fundus imaging, alongside specific laboratory analyzers for Hb measurement. Despite their reduced error, their correlation coefficients were relatively low, indicating weaker overall agreement between predicted and actual Hb values. While these studies [16,17,18,19,20] have contributed significantly to the field, many rely on specialized equipment or controlled environments. In contrast, our approach utilizes standard facial videos captured under natural lighting conditions, eliminating the need for additional hardware. This method enhances accessibility and practicality, particularly in diverse real-world settings, and achieves a competitive MAE of 0.85 g/dL, underscoring its potential for widespread application.

## 5. Conclusions and Future Works

This study presents a new method to detect SpHb values from facial videos using the 3D regression models. Videos from 279 volunteers, including 126 males and 153 females, are used in the study. In addition to these videos, SpHb values are obtained and recorded from all volunteers using Masimo, and this dataset is preprocessed for this study. Herein, the dataset is randomly split using ratios of 0.64, 0.16, and 0.20 for training, validation, and test sets, respectively. Using the training and validation sets, 3D CNN, channel attention-enhanced 3D CNN, and residual 3D CNN regression models are developed in the study. Performance metrics are calculated for all these models and a user-friendly GUI is designed to include the most successful model. To the best of our knowledge, this study is the first to directly estimate SpHb from facial videos using a 3D CNN-based video regression model. The limitation of this study is that it only detects videos and SpHb values obtained from healthy volunteers. In future studies, regression and classification architectures will be developed by expanding the range of SpHb values and using more models.

Compared to existing non-contact Hb estimation approaches based on smartphones, conjunctival images, or PPG signals, the proposed 3D CNN model offers a contactless, scalable, and low-cost alternative with competitive performance (MAE: 0.850 g/dL). As detailed in the updated Table 4, the model outperforms many methods using more constrained or specialized input modalities, while maintaining practical usability with simple video capture setups.

A key limitation of this study is the restricted dataset, which includes only healthy participants within a limited Hb range (11.2–16.9 g/dL), and an imbalanced gender distribution. Furthermore, variations in lighting, skin tone, and facial motion were minimized during data collection to reduce external noise; hence, the model’s robustness under real-world conditions could not be assessed.

In future work, we plan to expand the dataset by including participants with a broader range of Hb levels, more balanced demographic representation, and diverse skin tones. In addition, we aim to validate our system against gold-standard laboratory Hb analyzers in addition to pulse oximeters, ensuring clinical-grade accuracy. Lighting conditions will also be systematically varied to evaluate the sensitivity of the model to illumination changes. Additionally, new experiments will be designed to test the effects of facial dynamics such as expressions and head movement, which were intentionally excluded from the current dataset. Explainable AI methods such as attention maps and saliency visualizations will be investigated to explore which facial features contribute most significantly to Hb estimation. Due to the requirement for a more heterogeneous dataset and model adaptation, this line of work will be pursued as a separate follow-up study focused specifically on interpretability and model transparency.

## Figures and Tables

**Table 1:** Number of individuals below and above the anemia threshold (12 g/dL).

Anemia Status	Count
<12 g/dL	116
≥12 g/dL	163

**Table 2:** Training parameters for all models used in the study.

Training Options	Baseline, 3D Attention, and Residual Regression Models
Number of frames used (per sample)	224 frames
Input tensor shape	224 frames, 224 height, 224 width, 3 channels
Batch size	4
Number of epochs	100
Optimizer	Adam
Early stopping criterion	Validation loss < 0.3
Loss function	MSE

**Table 3:** Evaluation metrics obtained for the 3D regression models proposed in the study.

Model	Performance Metrics						
	MSE	R^2^	PCC	RMSE	MAE	Explained Variance	MAPE (%)
3D CNN (baseline) [29]	3.2029	−0.4687	0.5744	1.7897	1.4269	0.2871	10.90
Channel Attention-Enhanced 3D CNN [29]	1.3767	0.3972	0.6563	1.1733	0.9630	0.4022	7.81
Residual 3D CNN	1.1300	0.5000	0.7300	1.0600	0.8500	0.5100	6.00

**Table 4:** Comparison with recent invasive studies.

Reference	Methodology	Data Type	Dataset Size	Performance Metrics
[16]	EGE-UNet for eyelid segmentation and DHA(C3AE) for Hb prediction	Smartphone images of the eye	1124 perioperative eyelid images	MAE: 1.34 g/dL
[17]	Mask R-CNN for image segmentation and MobileNet for Hb prediction	Images of conjunctiva, palm, and fingernails	3705 images (1235 patients: eye, palm, nail image each) + 101 patients (prospective set)	Accuracy: 84.9% in anemia detection
[18]	InceptionV3 DL model	Retinal fundus images	114,257 fundus images (57,243 participants)	AUC: 0.88 in anemia detection
[19]	VGG16, ResNet50, and InceptionV3 architectures	Retinal fundus images	4517 fundus images (2265 participants) + 255 UWF images (external test set)	MAE: 0.58 g/dL
[20]	XGBoost regression model	Four-wavelength PPG signals from fingertips	PPG signal from 58 people in 4 wavelengths (160 features extraction)	MAE: 0.325 g/dL
Proposed System	3D CNN-based regression	Facial videos	Facial videos and synchronous Hb measurements from 279 participants	MAE: 0.850 g/dL

## Data Availability

The data that support the findings of this study are available from the project supervisor (ufukbal@osmaniye.edu.tr) upon reasonable request.
